# Barriers and facilitators to successful implementation of sustainable school meals: a qualitative study of the OPTIMAT™-intervention

**DOI:** 10.1186/s12966-021-01158-z

**Published:** 2021-07-03

**Authors:** Patricia Eustachio Colombo, Liselotte Schäfer Elinder, Emma Patterson, Alexandr Parlesak, Anna Karin Lindroos, Susanne Andermo

**Affiliations:** 1 Department of Global Public Health, Karolinska Institutet, 171 77 Stockholm, Sweden; 2 Solnavägen 1E, 11365, Stockholm, Sweden; 3 Centre for Epidemiology and Social Medicine, Region Stockholm, 113 65 Stockholm, Sweden; 4 The Swedish Food Agency, 751 26 Uppsala, Sweden; 5 Baden-Wuerttemberg Cooperative State University, 74076 Heilbronn, Germany; 6 Department of Nutrition, Exercise and Sports, University of Copenhagen, 1165 Copenhagen, Denmark; 7 Department of Internal Medicine and Clinical Nutrition, the Sahlgrenska Academy, University of Gothenburg, 405 30 Gothenburg, Sweden

**Keywords:** Sustainable diets, Public sector meals, Children, Implementation research, Public health

## Abstract

**Background:**

There is an urgent need to align human diets with goals for environmental sustainability and population health. The OPTIMAT™-intervention study was developed to implement and evaluate a nutritionally adequate and climate-friendly 4-week lunch menu in Swedish primary schools. This study aimed to explore pupils’ and kitchen staff’s experiences of the intervention and to identify barriers and facilitators to successful implementation of sustainable school meals.

**Methods:**

An inductive manifest qualitative method was used. Nine focus group discussions (FGDs) were conducted, six with pupils in grades 5 (ages 10–11) and 8 (ages 14–15) (*n* = 29) and three with kitchen staff (*n* = 13). Data were analyzed using qualitative content analysis.

**Results:**

Five main categories and 11 subcategories at a manifest level emerged. The five main categories were: 1) *Experiences with the new menu*, unfolding variations in how the new menu was received and kitchen staff’s experiences of working with it*;* 2) *The meaning of diet sustainability*, comprising pupils’ and kitchen staff’s perceptions about diet sustainability as a concept and part of their everyday lives; 3) *Factors influencing plant-based food acceptance*, covering aspects such as the influence of sensory factors, habits and peer pressure; 4) *Opportunities to increase plant-based eating*, including factors related to pupils’ and kitchen staff’s ideas for how to increase plant-based food acceptance*;* and 5) *Need for a supportive environment to achieve dietary change*, comprising pupils’ and kitchen staff’s thoughts on the importance of more knowledge, resources and involvement of stakeholders to eat more plant-based meals in schools.

**Conclusions:**

Successful implementation of sustainable school meals would require more knowledge among pupils and kitchen staff. Staff also need more training in cooking of sustainable meals. Barriers among pupils could be tackled by introducing new plant-based meals more gradually and by more carefully considering the seasoning, naming and aesthetics of dishes. An increased leadership support for change and involvement of stakeholders from multiple levels within society will be key in the transition to sustainable school meals at scale.

**Trial registration:**

The trial registration for the OPTIMAT™-intervention may be found at clinicaltrials.gov (NCT04168632 Fostering Healthy and Sustainable Diets Through School Meals (OPTIMAT)).

**Supplementary Information:**

The online version contains supplementary material available at 10.1186/s12966-021-01158-z.

## Introduction

There is an urgent need to align human diets with goals for environmental sustainability and population health. Suboptimal diets, low in whole grains, legumes, fruits and vegetables, and high in red and processed meat, sugar and salt are increasingly contributing to mortality and morbidity from chronic diseases in many regions of the world [1]. Current food demand is also perpetuating food production- and supply systems with detrimental climate impacts [2]. Adopting sustainable diets —"with low environmental impacts which contribute to food and nutrition security and to healthy life for present and future generations “[3]*—*is thus a pressing imperative to take action. In order to achieve the necessary dietary shifts, an understanding of the factors affecting food choices in different settings is needed to design effective interventions that may be able to change unsustainable dietary patterns [4].

School meals make up a considerable proportion of children’s dietary intake over a critical period of life, a period when core dietary habits are shaped that may persist throughout adulthood [5, 6]. School meals have thus been identified as an opportunity to effectively improve both health and environmental sustainability as they can reach most or all children depending on countries’ school-meal system and level of subsidy [6, 7]. In Sweden, nearly 200 million meals are served in all of the country’s almost 5000 primary schools (grades 0–9, ages 6–16) every year [8]. Globally, the country is currently one of few in providing lunches to all primary school children free of charge to parents, regardless of family income [9]. On average, about a quarter to a third of primary school children’s daily energy intakes is provided by the school lunch and approximately 70% of pupils eat the school lunch on all school days [10]. Thus, the reach and scale of the Swedish school meal system not only offers an important opportunity to reduce the climate impact of diets, but also to foster healthy and environmentally sustainable dietary habits in all children in both the short and long-term [6]. Based on this, the OPTIMAT™-project [11] was developed with the overall aim to optimize school meals in Sweden with regard to several dimensions of diet sustainability: nutritional content, greenhouse gas emissions (GHGE), affordability and acceptability to children. It was hypothesized that these optimized school meals would lead to consumption of more nutritious lunches with a lower environmental impact, without increasing waste and thus contribute to more effective use of public resources. To test this hypothesis, an intervention study was carried out during the spring of 2019 [12]. A new school lunch menu was developed which was 40% lower in GHGE, nutritionally adequate, 11% cheaper than the original, yet still similar in terms of composition. For example, in this new menu, only six of the 40 served dishes were entirely lacto-ovo vegetarian (i.e., did not contain any red meat/fish/poultry), compared to four in the usual (baseline) menu. The new menu was prepared and delivered by school kitchen staff for pupils in grades 0–9 [12]. The implementation of the new menu was deemed successful since the new menu did not increase food waste or decrease consumption. The children also anonymously answered a questionnaire about meal satisfaction, levels of which remained unchanged.

In order to roll out a program such as OPTIMAT™ more broadly, implementation needs to be prepared carefully [13]. Factors (e.g. barriers and facilitators) with the potential to affect the process in general need to be understood [14, 15]. These could be factors such as characteristics of the intervention itself such as the complexity and adaptability of the intervention [15]. There could also be barriers and/or facilitators in the inner setting of an organization (e.g. availability of resources) or in the outer setting (e.g. external policies and incentives) or in the implementation process [14]. The motivation to shift to more sustainable diets is more pronounced in younger than in older people in Sweden, where about a third of all young people are currently positive to consuming a more plant-based diet [16]. But there is currently limited insight into why and how these preferences develop. Understanding social norms relating to sustainable eating would thus also be essential as they can act as determinants of the adoption of sustainable diets [17]. Furthermore, few initiatives have so far implemented sustainable meals in the school context [18, 19] and research investigating determinants of implementation of sustainable school meals is limited.

The aim of this study was to explore pupils’ and kitchen staff’s experiences with the introduction of a nutritionally adequate and climate-friendly 4-week school lunch menu and to identify barriers and facilitators to successful implementation of sustainable school meals.

## Methods

### Study design

This study employed an inductive qualitative design to explore pupils’ and kitchen staff’s experiences with a school lunch intervention— the OPTIMAT™-intervention [12]— as well as to identify barriers and facilitators to successful implementation of sustainable school meals.

Focus group discussions (FGDs) were used to explore the experiences and perceptions of pupils and kitchen staff in the schools where the OPTIMAT™-intervention had taken place. Such qualitative methodologies are suitable when intending to investigate perceived barriers and facilitators to the implementation of interventions and enables an in-depth exploration of selected aspects [20]. Furthermore, FGDs are useful when the aim is to obtain an understanding about experiences and perceived usefulness of a phenomenon shared by a group [21, 22] such as, in this case, kitchen staff and pupils affected by the intervention. Group discussions were thus assumed to provide an opportunity for kitchen staff and pupils to compare and discuss their experiences and perceptions.

### The OPTIMAT™-intervention

The OPTIMAT™-intervention has previously been described in detail [12]. Briefly, the intervention consisted of developing the best possible (i.e. optimized) alternative 4-week school lunch menu that was 40% lower in GHGE, nutritionally adequate, and as similar as possible to the baseline school food supply in terms of food composition. The optimized menu was introduced in three schools (grades 0–9) in one Swedish municipality. A pre-post study design (with schools serving as their own controls) was employed to assess the effects of implementing the new lunch menu on daily food waste, consumption, and pupils’ school meal satisfaction. A 4-week measurement period, during which the baseline menu was served, preceded the 4-week intervention period during which the new menu was served. The school kitchen staff prepared and served the new menu, which contained ~ 32% less meat, 13% less dairy products, and ~ 7% more vegetables (including pulses and roots) as compared to the baseline menu. The information to pupils about the menu change was kept to a minimum to avoid potentially generating negative reactions that would influence the outcomes (food waste and consumption). Also, no social or pedagogical components were directed at the pupils, so that we could study the effect of changing the meals only.

### Setting and participants

The schools were located in an outer Stockholm municipality with around 93,000 inhabitants of which a high proportion are foreign-born. The average proportion of pupils with a non-Swedish background, i.e. pupils born abroad or born in Sweden with both parents born abroad, was 52% in the municipality (cf. 29% in Stockholm County and 26% nationally), while the average proportion of pupils with parents without a post-secondary education was 51% in the municipality (cf. 33% in Stockholm County, and 40% nationally) [8]. In the participating schools, the percentage of pupils with a non-Swedish background was 26% in School 1, 82% in School 2 and 75% in School 3.

The proportion of pupils with parents without a postsecondary education was 42% in School 1, 70% in School 2) and 60% in School 3. The same lunch menu is provided to all the municipality’s schools, but each school chef has some degree of freedom to adapt the menus slightly to match the preferences of their pupils.

Kitchen staff and pupils in grades 5 (ages 10–11) and 8 (ages 14–15) from the three schools where the intervention took place constituted the study participants. Grades 5 and 8 were selected to allow for a comparison with findings from the questionnaire used in the intervention [12] that covered indicators/dimensions of school-meal satisfaction in these grades. It was deemed crucial to understand the intervention through the eyes of the providers (kitchen staff) and the recipients (pupils) of this intervention. Hence, both groups were targeted to obtain a multifaceted and in-depth understanding of how the intervention was received and could be scaled up successfully.

Sampling for qualitative studies depends on what the researcher wants to know, the study’s purpose and available time and resources [20]. Considering the study aims, all 14 members of the kitchen staff with experience of having worked with the preparation of meals in the intervention were purposively selected to participate in the FGDs [20]. As for the pupils, class lists containing only information on names (from which sex was inferred) of all pupils in grades 5 and 8 in the intervention schools were obtained from the school administration. Based on this information, pupils were also purposively selected to achieve an equal distribution of males and females in the sample of pupils. No other information about pupils was available. The grades were not mixed in the FGDs as homogeneous groups are recommended when working with children [23]. Eight pupils in grade 5 and eight pupils in grade 8 in each of the three schools, (24 pupils per grade, 48 in total), were invited to participate in the FGDs through an invitation letter directed to both the pupil and legal guardian(s). School teachers in grades 5 and 8 disseminated these letters to pupils during class and instructed them to hand them over to their parents/legal guardians, obtain their signature and bring the letter with them back to school. Invited pupils consequently needed to show up at the FGD occasion and provide a written informed consent (signed by both parent and pupil) in order to be included in this study.

### Data collection

In total, nine FGDs were carried out in order to capture the perceptions and experiences of kitchen staff and pupils regarding the intervention and the concept of sustainable development [21]. Three FGDs were carried out with the kitchen staff (*n* = 13)—one FGD in each of the three schools—after lunch hours in the school canteen. Six FGDs were carried out, three with pupils in grade 5 and three with pupils in grade 8. From the 48 invited pupils, seven in grade 5 and seven pupils in grade 8 did not show up at the interview occasion and four in grade 5 and one in grade 8 did not provide a signed consent form. These 19 individuals were not included, leaving 29 pupils who agreed and provided written consent to participate in the study (see Supplemental Table 1 in Addition file 1 for further details on the FGDs and selection process).

The FGDs with pupils were held during school hours in a room, which was selected by teachers or principals. In two of the schools, FGDs were held in the school library, while one school chose the school canteen as the place for discussion. FGDs with kitchen staff and pupils were held during May 2019. One of the invited head chefs could not participate in the group discussion and was interviewed separately in August 2019.

A semi-structured interview guide was devised including predetermined topics with open-ended questions as well as complementary questions to enable clarification and exploration of answers in greater detail [24]. The following topics, intended to capture kitchen staff’s and children’s perceptions, thoughts, preferences and ideas about the intervention and sustainable development, were addressed during the discussions:

1. General perceptions regarding the school lunch/working in the school canteen.
2. Experiences of receiving/implementing the intervention.
3. Perceptions and views on sustainable development/sustainable diets, with focus on exploring opportunities and limitations to achieving a broad scale acceptability of more plant-based school meals.

The participants were encouraged to talk to each other rather than addressing the moderator [21]. One of the authors (PEC) acted as moderator for the discussion, and another author (SA) acted as an observer and took notes, but also occasionally asked complementary questions. None of them had been involved in the delivery of the intervention (i.e. in preparing or serving of the new menu). However, PEC was part of the research team that had developed the study. Only respondents and interviewers were present in the room during the discussions. The FGDs with kitchen staff lasted for 52 to 75 min, the separate individual interview with one of the kitchen chefs lasted for 29 min, while the discussions with pupils lasted for 24 to 50 min. All discussions were conducted in Swedish and audio was recorded.

### Data analysis

The recorded interviews were transcribed verbatim (by PEC) and analysed using inductive manifest qualitative content analysis [25, 26]. In the first step, the transcripts were read through several times to obtain a sense of the whole content. In this step, an initial open coding was made manually as described by Elo and Kyngäs [26]. In the second step, content that was found to be linked to the research questions (i.e. meaning units) was abstracted and condensed. Each condensed meaning unit was then assigned a code (comprising one or a few words) that contained the essence of the condensed meaning unit. Several meaning units could be given the same code if suitable. In the third step, all codes were compared and structured and consequently arranged into the categories and subcategories that emerged [25, 26]. Quotes that were chosen for the results-section were translated from Swedish to English. The translation was first made by a native Swedish speaker (PEC) and then verified by a native English speaker with full command of the Swedish language (EP).

The group discussions with the kitchen staff and pupils were initially analyzed separately to detect potential differences between the narratives of the two groups. As many commonalities emerged, the analyses were integrated and treated as an entity. However, the separate analyses were useful to provide descriptions of aspects that were not shared between the kitchen staff and pupils. The first step of the analysis was performed by PEC and SA, and the second and third step of the analysis was performed by PEC, who iteratively re-assessed the code-subcategory-category groupings through discussions with SA. During the process of analysis the categories and subcategories were defined through intersubjectivity between PEC and SA [25]. All authors provided input to the analysis after this step.

## Results

Five main categories and eleven subcategories emerged in the qualitative content analysis of the FGDs with pupils and kitchen staff (Table 1).

**Table 1:** Main categories and subcategories

Experiences with the new menu	The meaning of diet sustainability	Factors influencing plant-based food acceptance	Opportunities to increase plant-based eating	Need for a supportive environment to achieve dietary change
*Variations in how the new menu was received*	*A broad and varied understanding of diet sustainability*	*The decisive role of taste, appearance, smell and recognition*	*Focusing on familiar foods and naming dishes carefully*	*More knowledge, resources and inspiration*
*A challenging experience to work with the new menu*	*Diet sustainability important but hard to realize*	*Habits, peer pressure and fears challenging acceptance*	*Increasing exposure, normalisation and motivation*	*Increased stakeholder involvement*
			*Gradual and realistic changes are key*	

### Experiences with the new menu

#### Variations in how the new menu was received

Pupils and kitchen staff experienced the new menu in different ways. Some pupils had not noticed the change in menu while others had registered small changes such as new dishes and more vegetarian food:

“*I didn’t notice much, I just noticed that there was more vegetarian food and stuff like that.*” (Pupil grade 5, male)

They expressed that most food tasted better during the intervention period and that it was a positive experience to try new dishes. However, some of the pupils could recall that their peers had been dissatisfied with the food. Similarly, the kitchen staff experienced that pupils were often complaining about the food and that that they did not eat as much as usual during the intervention. .

Teachers were the ones perceived to have been complaining the most about the new menu according to the kitchen staff, even in front of the pupils. But there were also some dishes that the kitchen staff themselves were skeptical towards:

*“It tasted good, but the way it [the food] looked when you had followed the recipe, it was like ‘It can’t be served because no one will eat it’, I wouldn’t even have wanted to, if I just went in [to the canteen] and saw it, I wouldn’t have wanted to taste it.*” (Kitchen assistant, School 1).

#### A challenging experience to work with the new menu

Kitchen staff expressed that it was a challenging experience to work with the new menu during the intervention. Time, budget, palatability and management of leftovers were dominating aspects here. It was also considered fun to try new recipes, as well as interesting, but time-consuming to measure the kitchen and plate waste daily as required during the intervention. The new menu was perceived to be cheaper and influenced their thinking on how they could contribute to more sustainable practices:

*“You [started to] think a lot more … a lot more about things like economical aspects … but also about the environment, so you started thinking more, you did it automatically.”* (Head chef, School 2)

However, it was also challenging to handle leftovers during the intervention, especially from mixed dishes with pulses that they perceived as less appropriate to store and re-serve on another occasion because they would lose their palatability if re-heated. Several of the dishes in the new menu were also much more time-consuming to prepare and make tasty and appealing, which was considered to be another challenge. To tackle this, the kitchen staff needed to plan and work more with the seasoning of the dishes.

### The meaning of diet sustainability

### A broad and varied understanding of diet sustainability

The perception of the meaning of diet sustainability was broad and encompassed many different aspects. Among the kitchen staff, the concept of sustainability implied improvements in behaviors such as consumption patterns. They also discussed the concept of diet sustainability in relation to time and impact:

*“You cannot do something that lasts only for a week, but it should last for the rest of your life, [ … ] it should work and be seen somewhere, and be felt.”* Head chef, School 3

Furthermore, kitchen staff described diet sustainability as eating foods that are good for the whole body, organic food and foods that have a long shelf life (the Swedish word for sustainability is the same as “long-lasting”). Pupils discussed diet sustainability in relation to meat-consumption and food waste, which they thought were unsustainable practices. Pupils also talked about the co-benefits of environmentally friendly foods with respect to human health, stating that meat is not good for health while vegetables are health promoting. Other kitchen staff and pupils did not really know what diet sustainability meant to them.

### Diet sustainability important but hard to realize

The kitchen staff acknowledged the importance of considering sustainability in their day-to-day practice (before the intervention) and thus tried to contribute to environmental sustainability when ordering food, e.g., exploring ways to make substitutions that are better from a climate perspective. The economic aspect was nevertheless perceived as a limitation in making decisions (before the intervention) as the kitchen staff have a specific budget that they need to stick to.

Pupils also recognised the need to prioritize sustainability and take responsibility for achieving sustainable development. They thought that eating less meat (due to its contribution to the emissions of greenhouse gases and climate change) was one of many ways to contribute to sustainable development, although they did not at all highlight this aspect when describing the factors that determined their dietary choices. Pupils further expressed that this issue was hard to implement, despite a will to do so:

*“… you always try to think 'Yes, well I’ll do it', but to break these habits that you have all the time … Well, it’s surprisingly difficult. You’re only [living] in the present.”* (Pupil grade 8, female)

### Factors influencing plant-based food acceptance

#### The decisive role of taste, appearance, smell and recognition

Pupils expressed a general dislike of school meals, irrespective of the intervention, and they perceived it as not tasting very nice. How food tastes was thus highlighted as one important aspect to the acceptability of plant-based (i.e. vegetarian or vegan) foods and pupils thought that plant-based dishes should be seasoned better. The (less preferable) taste of plant-based dishes was thus expressed as an important reason for why they were currently not widely accepted amongst pupils:

*“Most people don’t like vegetarian dishes. So that's why they don’t eat.”* (Pupil grade 8, female)

The appearance of the food, its smell but also the pupils’ familiarity with different dishes, were also seen as important determinants of the acceptability of plant-based dishes. Pupils expressed that they preferred plant-based dishes that resembled animal-based foods, dishes that they recognized and appreciated:

*“...yes well, most people like chicken, so you can have something that is quite similar to chicken in terms of both taste and texture [to make it more acceptable].”* (Pupil grade 5, female)

#### Habits, peer pressure and fears affecting acceptance

Kitchen staff perceived that pupils in general have difficulties accepting plant-based dishes as they are not in the habit of eating mixed dishes with beans and vegetables. Peer pressure was also seen as a barrier to getting pupils to eat school meals in general, something that was seen as more likely to happen with unfamiliar dishes.

*“Sometimes four or five pupils can come [to the canteen] and then when one [of the pupils] says 'No we are not going to eat that', they just look at each other and then they just leave.”* (Head chef, School 1)

Pupils perceived that there is a general reluctance towards eating plant-based foods that inhibits them from eating/trying the school lunch.

*“People are kind of afraid to taste. It's usually not so disgusting, it's just that when it says that it's vegetarian, then people don’t even want to taste.”* (Pupil grade 8, male)

### Opportunities to increase plant-based eating

#### Focusing on familiar foods and naming dishes carefully

The kitchen staff perceived that there are several vegetarian dishes such as pasta dishes, vegetarian burgers and tortillas that the pupils like and thus could be served more frequently. Lentils could also be served more often since they are more widely accepted and easily mixed in with meat dishes where they are not directly visible to the children. Kitchen staff also said that it works well when they avoid the term “vegetarian” when naming dishes, and instead use names that pupils recognize:

*“Yes but lasagna, kids like lasagna. It’s the name lasagna they like. They eat lasagna even though it is 'green' [vegetarian].”* (Kitchen assistant, School 2)

In line with this, pupils talked about vegetables as something distinct from vegetarian food:

*“I don’t eat vegetarian very often, and when I eat it, well I prefer like normal vegetables over like vegetarian things and stuff like that. So, I would rather take like meat and vegetables, than vegetarian foods really.”* (Pupil grade 5, male)

#### Increasing exposure, normalisation and motivation

The pupils discussed increased exposure to more plant-based foods in the canteen and/or through school activities that could be key in making them more accepted. They further thought that plant-based eating should not be associated with the act of having to take a stand on something but thus instead be treated as normal practice:

*“Well, that it isn’t connected to anything that’s healthy or political … If we were to have vegetarian days, they should not be so connected to like 'Oh this is sustainable development', of course you have to be aware of it but there are people who know it anyway. And it should not be the healthy alternative but rather just something that’s common.”* (Pupil grade 8, female)

Another way of making plant-based foods more acceptable could be to highlight motives to why these foods could be beneficial to them on a personal level:

*“I think that people might become more interested if they realize that it [the change] can benefit them on a personal level ... That you learn what the positive consequences for yourself would be … that it might make a difference for your studies in school, that you feel better. There are many who play sports, and they could learn why it’s good for them, because everyone wants to get better at something, and by knowing why it can be better, then maybe people will be a little more inclined to, like, 'Yes well it wasn’t that hard'.”* (Pupil grade 8, female)

#### Gradual and realistic changes are key

Both pupils and kitchen staff seemed to agree on the fact that a dietary shift towards more plant-based food could be achieved by employing moderate, gradual changes. Pupils highlighted the importance of finding solutions to sustainable habits that are realistic:

*“I don’t know if you could remove the meat completely, but you really have to change [eating habits] for the sake of the environment, you have to think about what’s sustainable. You might not need to remove all meat and you could chose organically or environmentally friendly produced [foods], I don’t know … The solution is to find environmentally friendly ways that don’t requires us to give up everything.”* (Pupil grade 8, female)

Similarly, kitchen staff thought that menus like the one used in the intervention were too large a change for the children. They discussed the importance of making stepwise changes to the school meals instead. Furthermore, kitchen staff discussed the importance of making children accustomed to plant-based meals already in preschool in order to create a natural transition to the climate friendly lunches in school.

### Need for a supportive environment to achieve dietary change

#### More knowledge, resources and inspiration

In order to achieve dietary changes towards more plant-based eating in schools on a broad scale, both pupils and kitchen staff brought up the need for a supportive environment with more knowledge of why and how to eat (and cook) more sustainably. Although the school curriculum includes some aspects about sustainable eating, pupils still thought that there is a need to discuss this issue more. Kitchen staff said that they completely lack training in sustainable cooking. They also stated that they lack financial resources and adequate equipment in their work environment to have the capacity to produce sustainable meals of good quality. Educational initiatives that can promote inspiration and motivation among the staff were seen as something that can create positive attitudes around the food that in turn can spread to the pupils. One of the head chefs highlighted the importance of being inspired and motivated in order to be able to influence diners

“*You could serve carrots in broth - if you think it's super tasty, then you can make diners think so too...”* (Head chef, School 3).

#### Increased stakeholder involvement

The aspect of involvement was in different ways highlighted as an important factor to achieving more plant-based eating in schools. For example, pupils said that they eat more at home where they get to decide more. They thus thought that they should be more involved in deciding the menus, but that they are currently not listened to:

*"Sometimes they say something like 'What foods would you like? Write a wish-list and then we’ll do it'. We write a wish- list, but nothing happens.”* (Pupil grade 8, male)

Many schools in Sweden have school food councils that are composed of representatives from pupils within each school. Kitchen staff considered the (existing) school food councils as important as they provide inputs and new ideas for dishes and menus. Kitchen staff also thought that they themselves need to be more involved in developing new menus, making use of their knowledge about the context, their experience and creativity, to develop dishes that better match their dining guests’ preferences. An increased involvement from, and cooperation between kitchen staff, teachers, the school management, parents, and politicians in the municipality were also seen as critical to achieving broad-scale dietary change towards more plant-based eating in schools.

## Discussion

In this study, we have explored pupils’ and kitchen staff’s experiences of the introduction of a 4-week GHGE-reduced school lunch menu, as well as barriers and facilitators to a future successful implementation of sustainable school meals. Five categories at a manifest level emerged: one describing pupils’ and kitchen staff’s experiences of the new menu, and four describing aspects of relevance to the implementation process. These four categories describe several barriers, concerning e.g., current habitual eating behaviors and resource limitations, that could impede successful implementation of sustainable school meals. They also describe opportunities such as increased exposure to plant-based foods and an increased involvement from stakeholders, that in turn could facilitate the implementation process.

The results imply that kitchen staff and pupils experienced the OPTIMAT™-intervention differently. While pupils did not seem to have noticed major changes to the menu, kitchen staff found it challenging to work with the new menu at times. For example, several of the dishes in the new menu were experienced as more time consuming to prepare and they had to dedicate more efforts than usual into making the meals tasty and appealing. This could be seen as a barrier since the implementation of a new program should ideally not be more complex than the procedures already in place [27]. Accordingly, school programs that do not require too large deviations from existing activities are more easily implemented into routine practice [28]. But simple changes can also be problematic since they instead might not be sufficiently extensive to deliver the desired effects [29]. In the case of implementing sustainable school meals effectively, the kitchen staff's perceived challenges with implementing the new menu could reflect a low compatibility [14] between the required changes and the current availability and/or prioritization of resources (including money, training, and time) in the inner setting of the school organization. This is further supported by the kitchen staff’s and pupils’ own statements through which they address the need for a supportive environment, including more training/educational activities, to achieve dietary changes towards more plant-based eating in schools. There is, in fact, evidence to suggest that interventions to improve children’s diets in schools are more likely to be successful when combining education with environmental changes [30, 31]. By incorporating training of kitchen staff and classroom activities for pupils focusing on sustainable development, an increased understanding of the relationship between food systems, health and the environment could translate into positive attitudes towards eating climate-friendly lunches. However, the realization of such opportunities is likely to require a strong leadership support, which is seen as critical to implementation [15]. A lack of leadership support has been acknowledged as a barrier to implementation for other school-based programmes [32]. Therefore, support from the school’s headmaster and stakeholders from multiple levels within society will be key in the transition to sustainable school meals at scale.

A new program is more likely to be implemented successfully when the need for it is recognized, there is a belief that the new program will be able to provide the desired gains, and when stakeholders have the necessary skills and a strong confidence in their capability to deliver on expectations (self-efficacy) [15]. In our case, the involvement from stakeholders, ranging from pupils and kitchen staff to decision makers in the municipality, was seen as key to achieving dietary changes towards more plant-based eating in schools. Research has demonstrated evidence of the benefits of emphasizing active engagement from the target group/involved stakeholders in the design and implementation of an intervention [33, 34]. For example, involving children in these processes has been shown to have positive effects on aspects such as their personal motivation, capabilities and awareness [34]. These were aspects that were also highlighted by the pupils who in the FGDs discussed both their own involvement and personal motivation as key to increasing plant-based food acceptance.

Familiarity with, and knowledge in relation to, the concept of sustainability among kitchen staff and pupils varied. Some of the kitchen staff expressed that they did not know what the concept of sustainability meant to them, while others elaborated their views on the concept and expressed that they tried to include the aspect of sustainability in their daily work. Pupils also demonstrated an understanding of key concepts and facts related to diet sustainability (e.g., the climate impact of meat), but they did not at all highlight this aspect as influential to their dietary choices. Despite knowing the benefits of eating plant-based foods, rather than animal foods such as meat, sensory factors such as taste, the food’s appearance and recognition were brought forward as determining factors in their choice of foods. This mirrors previous research where the lack of availability and variety of preferred foods [35] and undesired aesthetics of foods [36] have been identified as barriers to school food acceptance amongst children.

Assuming that children will adopt sustainable behaviours just by being provided with the relevant information can thus be limiting since children’s eating behaviours are likely to be affected by a complex interaction between biological, environmental, cultural, and social factors [37]. Hence, having the right knowledge and beliefs [14] surrounding these aspects might not be the most critical factor to succeed in increasing plant-based food acceptance. The likelihood of pupils accepting initiatives like the OPTIMAT™-intervention might be more likely to depend on the extent to which the offered solution aligns with their habitual eating behaviours formed over the continuum of life (within individuals, families, peers or other social constellations), and their sociocultural heritage [38]. In the FGDs with kitchen staff, pupils' habitual eating patterns were considered to be a barrier to plant-based food acceptance. At the same time, the interviews also revealed another side of the coin. By increasing exposure to plant-based dishes that the pupils recognize—dishes that they like and are familiar with respect to appearance and seasoning (rather than completely new ones)—and by using names that they recognize, existing eating habits were also seen an opportunity to increase acceptance of plant-based food in schools. Both kitchen staff and pupils also underlined the opportunity for making plant-based eating part of normal practice by increasing exposure to non-habitual plant-based foods gradually, and preferably as early as in pre-school. This is aligns well with research showing that increased exposure is a factor known to positively influence acceptance of new foods among children [39]. Capitalizing on both opportunities (i.e. increased exposure to *both* habitual and novel plant-based dishes) could thus have potential to enhance plant-based food acceptance in schools as well as in the home environment [38, 40].

The results from the outcome evaluation of the OPTIMAT™-intervention [12] show that kitchen staff followed the new menu as planned with the exception of minor changes that kitchen staff were allowed to make to recipes when preparing the meals (e.g., changing seasoning, or one type of bean for another due to availability). Thus, fidelity to the intervention was high. Furthermore, there were no observed changes in food waste or consumption, which indicates an unchanged school meal acceptance in this intervention [12] and in another comparable study [41]. In contrast, kitchen staff’s experience was that pupils were complaining more about the food when the new menu was served. This discrepancy could reflect pupils’ general dislike of the school meal, irrespective of the intervention, something that also became evident from the pupil-questionnaire on school meal satisfaction and the fact that about 20% of all prepared food was wasted during both baseline and intervention periods [12]. Kitchen staff's perceptions of the pupils’ negative reactions to the new menu could thus be mirroring the fact that they were more attentive towards pupils' daily feedback during the intervention period. Pupils’ general dislike towards school meals could be a barrier for successful implementation of sustainable meals following the approach used in OPTIMAT™ [12]. Here, the new menu was developed to be as similar as possible to the ordinary menu. Yet, if ordinary menus are not widely accepted in the first place, new sustainable menus are also likely to struggle to reduce food waste, one of the UN sustainable development goals [42]. Future efforts should therefore aim to explore and integrate pupils' preferences in depth and consider these more extensively in both development of new school meals and during implementation.

To the best of our knowledge, this is the first qualitative research study to focus on implementation barriers and facilitators of sustainable school meals. The study employed a multi-stakeholder approach by exploring the experiences and perceptions of both intervention providers (kitchen staff) and its recipients (pupils), thus increasing its credibility [20]. Our understanding of how to successfully implement sustainable meals in schools could be improved by also including the perspective of teachers in this qualitative assessment. Teachers act as important role models to pupils, both in the classroom and in the canteen. Hence, their perceptions and attitudes are likely to be of importance to successful implementation of sustainable school meals and would be valuable to explore in future studies.

It is a limitation of this study that views of kitchen staff and pupils from three schools in one Swedish municipality might not be sufficient in establishing the desired trustworthiness of our research findings [43, 44]. Even though we were able to include the experiences and perceptions of all kitchen staff involved in the intervention, the transferability of our findings might partly be limited since barriers and facilitators to successful implementation of sustainable school meals may be very contextual and show great variation, especially with regards to e.g., local policies, norms, and preferences. Furthermore, we only invited a limited group of pupils exposed to the intervention (from grades 5 and 8 only) to participate in the FGDs. From these, almost 40% could not be included in this study as they failed to either show up or provide written consent at the FGD occasion. There is a possibility that the pupils that were part of the FDGs shared common views and experiences that differed from those who were not included. Therefore, our findings may not be able to provide a sufficiently complete understanding of how the intervention was experienced by pupils in general or in other municipalities. The credibility of our findings should thus be interpreted with this in mind. However, there are also several aspects that may be considered to strengthen the trustworthiness of our findings. This includes both the investigator triangulation performed by involving all authors in addressing organizational aspects related to both the planning and execution of the study [43, 44]. Furthermore, data were analysed and discussed by two different researchers (PEC and SA) and persistent observation of the data (i.e. giving particular attention to the characteristics and aspects of a situation that are relevant to the phenomena being pursued) was undertaken to enable further triangulation [43]. Besides the presented physical audit trail (realized by describing methodological stages and decisions of this study), an intellectual audit trail (self-reflection on e.g., personal assumptions/preconceptions in relation to research decisions) was also prioritized (by PEC) to augment the trustworthiness of the study [45].

## Conclusions

Our findings highlight discrepancies in how the OPTIMAT™-intervention was experienced by pupils and kitchen staff while also identifying both barriers and facilitators to successful implementation of sustainable school meals. Pupils’ habitual eating patterns may act as both a barrier and facilitator to acceptance of more plant-based foods and are thus important to consider. Other important barriers that were encountered concern personal factors such as the role of sensory factors in pupils' food choices, pupils' general dislike of school meals, and the lack of involvement of stakeholders (from pupils to decision makers in the municipality) in the change process. From an implementation perspective, these barriers could be tackled by introducing new plant-based meals more gradually, and by carefully considering the seasoning, naming and appearance of dishes. Leadership support should be provided, and resources mobilized so that pupils and kitchen staff can receive education in sustainable diets. Moreover, kitchen staff need training in the production of more plant-based meals. In addition, we need a better understanding as to why pupils in general have negative attitudes towards school meals before introducing new menus that are optimized and planned to mimic current meals. Lastly, involvement from a wide range of stakeholders from pupils and kitchen staff to decision makers in the municipality is likely to be key in achieving a transition to more climate-friendly school meals at scale.

## Supplementary Information

**Additional file 1 Supplemental Table 1**. Details on the inclusion of participants for the focus group discussions.

## Data Availability

Data can be made available from the corresponding author upon reasonable request.

## Abbreviations

FGDs: Focus group discussions
GHGE: Greenhouse gas emissions
