# Supplementary material for: Barriers and facilitators to successful implementation of sustainable school meals: a qualitative study of the OPTIMAT™-intervention
Source: Int J Behav Nutr Phys Act. 2021 Jul 3;18:89. doi: 10.1186/s12966-021-01158-z (PMC8254978; doi:10.1186/s12966-021-01158-z)
Supplement: Supplementary file 1 — Additional file 1 Supplemental Table 1. Details on the inclusion of participants for the focus group discussions. [file 12966_2021_1158_MOESM1_ESM.docx]

**Supplementary Table 1.** Detailed information on the number of individuals available for recruitment to the focus group discussions in each school, the number of invited individuals and the number of persons participating, according to discussion group (kitchen staff versus pupils), sex and pupils’ grade.

|  | **School 1** | | |  | **School 2** |  | **School 3** | | |
| --- | --- | --- | --- | --- | --- | --- | --- | --- | --- |
|  | Available | Invited | Participated | Available | Invited | Participated | Available | Invited | Participated |
| **School chef** |  |  |  |  |  |  |  |  |  |
| Male | 0 | 0 | 0 | 1 | 1 | 1 | 0 | 0 | 0 |
| Female | 1 | 1 | 1 | 0 | 0 | 0 | 1 | 1 | 1 |
| **Total** | 1 | 1 | **1** | 1 | 1 | **1** | 1 | 1 | **1** |
| **Kitchen assistants** |  |  |  |  |  |  |  |  |  |
| Male | 0 | 0 | 0 | 0 | 0 | 0 | 1 | 1 | 1 |
| Female | 5 | 5 | 5 | 3 | 3 | 3 | 2 | 2 | 2 |
| **Total** | 5 | 5 | **5** | 3 | 3 | **3** | 3 | 3 | **3** |
| **Grade 5 pupils** |  |  |  |  |  |  |  |  |  |
| Male | 17 | 4 | 2 | 15 | 4 | 2 | 21 | 4 | 2 |
| Female | 11 | 4 | 3 | 21 | 4 | 3 | 17 | 4 | 3 |
| **Total** | 28 | 8 | **5** | 36 | 8 | **5** | 38 | 8 | **5** |
| **Grade 8 pupils** |  |  |  |  |  |  |  |  |  |
| Male | 11 | 4 | 1 | 20 | 4 | 4 | 16 | 4 | 3 |
| Female | 17 | 4 | 3 | 25 | 4 | 1 | 10 | 4 | 2 |
| **Total** | 28 | 8 | **4** | 45 | 8 | **5** | 26 | 8 | **5** |
